# Discovery of Novel Biomarkers for Diagnosing and Predicting the Progression of Multiple Sclerosis Using TMT-Based Quantitative Proteomics

**DOI:** 10.3389/fimmu.2021.700031

**Published:** 2021-08-20

**Authors:** Yijun Shi, Yaowei Ding, Guoge Li, Lijuan Wang, Rasha Alsamani Osman, Jialu Sun, Lingye Qian, Guanghui Zheng, Guojun Zhang

**Affiliations:** ^1^Laboratory of Beijing Tiantan Hospital, Capital Medical University, Beijing, China; ^2^NMPA Key Laboratory for Quality Control of In Vitro Diagnostics , Beijing, China; ^3^Beijing Engineering Research Center of Immunological Reagents Clinical Research, Beijing, China

**Keywords:** multiple sclerosis, biomarker, proteomics, differentially expressed proteins, IGFBP7, SST

## Abstract

**Objective:**

Here, we aimed to identify protein biomarkers that could rapidly and accurately diagnose multiple sclerosis (MS) using a highly sensitive proteomic immunoassay.

**Methods:**

Tandem mass tag (TMT) quantitative proteomic analysis was performed to determine the differentially expressed proteins (DEPs) in cerebrospinal fluid (CSF) samples collected from 10 patients with MS and 10 non-inflammatory neurological controls (NINCs). The DEPs were analyzed using bioinformatics tools, and the candidate proteins were validated using the ELISA method in another cohort comprising 160 samples (paired CSF and plasma of 40 patients with MS, CSF of 40 NINCs, and plasma of 40 healthy individuals). Receiver operating characteristic (ROC) curves were used to determine the diagnostic potential of this method.

**Results:**

Compared to NINCs, we identified 83 CSF-specific DEPs out of a total of 343 proteins in MS patients. Gene ontology (GO) enrichment analysis revealed that these DEPs are mainly involved in platelet degranulation, negative regulation of proteolysis, and post-translational protein modification. Pathway enrichment analysis revealed that the complement and coagulation cascades, Ras signaling pathway, and PI3K-Akt signaling pathway are the main components. Insulin-like growth factor-binding protein 7 (IGFBP7), insulin-like growth factor 2 (IGF2), and somatostatin (SST) were identified as the potential proteins with high scores, degree, and centrality in the protein-protein interaction (PPI) network. We validated the expression of these three proteins using ELISA. Compared to NINCs, the level of CSF IGFBP7 was significantly upregulated, and the level of CSF SST was significantly downregulated in the MS group.

**Conclusion:**

Our results suggest that SST and IGFBP7 might be associated with the pathogenesis of MS and would be helpful in diagnosing MS. Since IGFBP7 was used to classify relapsing remitting MS (RRMS) and secondary progressive MS (SPMS) patients, therefore, it may act as a potential key marker and therapeutic target in MS.

## Introduction

Multiple sclerosis (MS) is a chronic autoimmune demyelinating disorder of the central nervous system (CNS). It is the main cause of severe neurological effects and disabilities in young adults (1). An estimated 2.5 million people worldwide are affected with MS, and it is more common in women than in men (2). In MS, symptoms exhibit a substantial degree of heterogeneity. Upon diagnosis, MS is classified into four types based on disease presentation, which include clinically isolated syndrome (CIS), relapsing remitting MS (RRMS), primary progressive MS (PPMS), and secondary progressive MS (SPMS). The majority of patients are affected with RRMS (85–90%); however, based on the statistics, over time, approximately half of these patients are prone to develop SPMS (3, 4).

Currently, the diagnosis of MS is mainly based on the clinical parameters and imaging examinations; however, human body fluid-based biomarkers are still largely unidentified. Although blood is the more readily available biofluid than cerebrospinal fluid (CSF), it is not the optimal sample for investigating the biomarkers in CNS disease because of the existence of the blood-brain barrier (BBB) (5). The detection of CSF markers can directly reflect the ongoing pathological and inflammatory processes due to the contact with the interstitial fluid in the CNS. Therefore, CSF markers, for example, the detection of oligoclonal bands (OB), immunoglobulin G (IgG) index, and 24 h IgG intrathecal synthesis rate have become the main research model in MS (6). However, these conventional CSF biomarkers exhibit comparatively low sensitivity and specificity, which restricts their application in the diagnosis of disease, assessment of prognosis, and treatment responses. In conclusion, it is imperative to identify novel biomarkers of MS.

Proteomics has been developed with the support of mass spectrometry and bioinformatics technology which provides a high-resolution, high-accuracy strategy to identify novel MS biomarkers (7, 8). In the present study, we aimed to discover the potential biomarkers through a comparative analysis of CSF and blood samples collected from MS patients and controls. For this study, we first used TMT labelling in combination with high-resolution LC-MS/MS analysis to reveal the differential proteome in CSF of MS patients and non-inflammatory neurological controls (NINCs). Then, the candidate biomarkers were validated using ELISA in a different cohort. This study helped to determine the molecular or pathophysiological processes related to MS by protein profiling, which also helped to discover novel biomarkers of MS.

## Materials and Methods

### Patient Samples

From 2020 to 2021, 140 participants who enrolled at the Beijing Tiantan Hospital of Capital Medical University were included in the present study. The study was conducted in two stages. Potential biomarkers were initially identified using a discovery cohort containing 10 patients with MS and 10 NINCs. Following this, the selected biomarkers were validated using a validation cohort containing 40 cases of MS patients (including 23 RRMS and 17 SPMS), 40 NINCs, and 40 healthy control (HC) cases.

Inclusion criteria for MS patients were as follows: (1) age≥18 years; and (2) diagnosis of MS according to the 2017 revised McDonald criteria (9). Exclusion criteria were as follows: (1) age<18 years; (2) serious diseases of the heart, liver, kidney, and other important organs or blood vessels; and (3) complication due to malignant tumor.

Our study was conducted in accordance with the Declaration of Helsinki and approved by the ethical committee of the Tiantan Hospital of Capital Medical University (Ethics Committee document number: KY 2020-076-02). All patients submitted written informed consent.

### TMT-Based Quantitative CSF Proteomics

#### CSF Protein Extraction and Digestion

Lumbar puncture was performed on all subjects after local anesthesia with lidocaine, and 3 mL of CSF was collected and stored into several 1.5 mL Eppendorf tubes at -80°C before use. Protein concentration was determined using the BCA assay after the removal of high abundance protein as instructed by the Proteominer Protein Enrichment Kit (Bio-Rad).

Enzymatic hydrolysis was conducted by using the FASP method (10). Briefly, 100 μg CSF of samples were taken from each group for enzymatic hydrolysis. Then, 200 μL of UA buffer (8 M urea, 150 mM Tris-HCl pH 8.0) was added and mixed, and the mixture was transferred to a 10 KDa ultrafiltration tube, and centrifuged at 12000 g for 15 min. Following this step, 200 μL of UA buffer was added and the tube was centrifuged at 12000 g for 15 min, and then the filtrate was discarded. Thereafter, 100 μL of IAA (50 mM IAA in UA) was added, and the tube was vortexed at 600 rpm for 1 min, incubated in dark at room temperature for 30 min, and centrifuged at 12000 g for 10 min. Following this, 100 μL UA buffer was added, and the tube was centrifuged at 12000 g for 10 min, and this step was repeated twice. After adding 200 μL of 100 mM TEAB, the tube was centrifuged at 12000 g for 10 min, and again, this step was repeated twice. Finally, 8 μL trypsin buffer was added and the tube was vortexed at 600 rpm for 1 min followed by incubation at 37°C for 16-18 h. A new collection tube was placed and centrifuged at 14000 g for 10 min, following which the filtrate was collected and freeze-dried.

#### TMT Labeling

Peptides from the CSF samples were labeled using TMT 10-plex™ Label Reagent set as per the manufacturer’s instruction (Thermo Fisher Scientific, USA). Briefly, before use, the TMT labeling reagents were removed from the freezer and stabilized at room temperature (approximately 30 min). Then, 41 μL of ACN was added to each channel, dissolved through vortexing, centrifuged, and set aside. The dissolved reagent was added to 100 μg of sample (i.e., mix 1:1). The solution was allowed to stand for at room temperature for 1 h. After adding 8 μL of hydroxylamine, the solution was incubated at room temperature for 15 min to terminate the reaction. The samples of each group from 10 channels were combined separately, and the vortexed to allow complete mixing. Salt and other impurities were removed from the sample before freezing at -80°C.

#### HPLC Fractionation

The peptides were fractionated using the UPLC 3000 system (Dionex, Sunnyvale, CA, USA) associated with an XBridgeTM BEH300 C18 column (Waters, Milford, MA, USA). Mobile phase A was H_2_O adjusted to pH 10 using ammonium hydroxide, while mobile phase B was acetonitrile adjusted to pH 10 using ammonium hydroxide. Peptides were separated using the following gradient: 8 to 18% phase B for 30 min, and 18 to 32% phase B for 22 min. A total of 48 fractions were collected, which were dried using a speed vac, combined to prepare a total of 12 fractions, and resuspended using 0.1% formic acid.

#### LC-MS/MS Analysis

All labeled tryptic peptides were separated by Easy nLC-1000 system coupled to the Q Exactive mass spectrometer (ThermoFisher Scientific). Peptides were separated on a 15 cm column (i.d. 75 um) packed in-house with the reverse-phase (RP) materials ReproSil-Pur C18-AQ, 3.0 um resin (Dr. Maisch GmbH, Germany). Data was acquired by Xcalibur in the data-dependent “top15” mode as follows: 15 most abundant precursor ions in each full scan (MS1 scan: 300-1500 m/z with resolution 120,000@ m/z 200, AGC target: 3E6, maximum IT: 50 ms) were selected by isolation window of 1.0 Da, and resolution for MS/MS spectra was set to 60000 @ m/z 200, target value was 1E5 (AGC control enabled, maximum IT: 50 ms), fragmentation mode of Higher Energy Collision Dissociation (HCD), normalized energy of 30%, and dynamic exclusion at 20 s.

All MS/MS spectra were analyzed using MaxQuant (9) (version. 1.6.0.16) to identify the peptides and proteins using the UniProt human fasta protein database (dated 202104, with 92607 protein items). TMT tags on lysine residues and peptide N termini (229.162932 Da) and carbamidomethylation of cysteine residues (57.02146 Da) were set as static modifications, while oxidation of methionine residues (+15.99492 Da) was set as a variable modification. Two missing cleavage site was allowed. The tolerances of peptides and fragment ions were set at 10 ppm and 20 ppm, respectively. The peptide and protein false discovery rate (FDR) was fixed at no more than 0.01. The reporter ion quantitation were based on the extraction of the TMT reporter ion signals for each peptide by MaxQuant software. In addition, to minimize the co-isolation interference, the precursor ion fraction (PIF) of 75% was set in MaxQuant as described elsewhere (11). Proteins were then quantified by summing reporter ion counts across all matching peptide matches, and normalized assuming equal protein loading across all samples.

#### Bioinformatic Analysis

Differentially expressed proteins (DEPs) were identified using 2-fold change and t-test corrected with the Benjamini-Hochberg FDR (*P<*0.05). Next, the proteins were analyzed using different bioinformatics tools. Heat map was plotted using the R software (12). Gene Ontology (GO) and Kyoto Encyclopedia of Genes and Genomes (KEGG) pathway enrichment analyses were performed for the identified DEPs using the “clusterProfiler” and “DOSE” packages in R software (13–15). Protein-protein interaction (PPI) network analysis was performed using the online database STRING (https://string-db.org/) (16). Cytoscape (v.3.6.0) software was used to visualize the resulting network (17).

#### Enzyme-Linked Immunosorbent Assay (ELISA)

A validation experiment was conducted using ELISA. Insulin-like growth factor-binding protein 7 (IGFBP7), insulin-like growth factor 2 (IGF2), and somatostatin (SST) concentrations were determined using CSF and plasma samples of the patients using the ELISA kit (ImmunoClone, USA). The experiments were performed in accordance with the manufacturer’s instructions.

#### Statistical Analysis

Continuous variables were compared using Student’s t test and Mann-Whitney U test depending on whether the data were normally distributed. Spearman’s correlation coefficient was used for the rank correlation analysis. The predictive value was assessed using the area under the receiver operating characteristic (ROC) curve. All tests were two-tailed. The data were analyzed using SPSS (version 22.0; IBM New York, USA) or GraphPad Prism 8 (GraphPad Software Inc., San Diego, CA, USA) softwares. For each statistical analysis, a *P* value less than 0.05 was considered as statistically significant.

## Results

### Clinical Characteristics of Patients

The clinical characteristics of 140 patients are shown in **Table 1**. There was no significant difference in terms of age or sex between the patients with MS and NINCs in the discovery group (*P*>0.05). Similarly, no significant difference was observed among the MS, NINCs, and HC groups in the validation group in terms of age and sex (*P*>0.05). MRI T2 lesion counts were also obtained from the medical records. Disability was scored by a qualified neurologist using the expanded disability status scale (EDSS) (18).

**Table 1:** Demographic and clinical data for the participates included in the study.

Variable	Discovery Cohort		Validation Cohort			
	MS (N = 10)	NINCs (N = 10)	MS (N = 40)		NINCs (N = 40)	HC (N = 40)
			RRMS (N = 23)	SPMS (N = 17)		
N, CSF; Serum	10; 0	10; 0	23; 23	17; 17	40; 0	0; 40
Age (year), mean ± SD	32.9 ± 10.4	41.9 ± 15.9	39.8 ± 14.0	33.1 ± 11.3	36.6 ± 9.9	38.2 ± 11.6
Male (%)	4 (40%)	6 (60%)	8 (34.8%)	6 (35.3%)	13 (32.5%)	14 (35%)
Disease duration (year), mean ± SD	4.3 ± 4.1	–	3.6 ± 3.8	14.2 ± 8.1	–	–
EDSS, mean ± SD	2.5 ± 1.2	–	2.8 ± 1.5	5.4 ± 1.2	–	–
MRI lesion		–			–	–
0-8 lesions, n, n%	4, 40.0%		13, 56.5%	7, 41.2%		
≥9 lesions, n, n%	6, 60.0%		10, 43.5%	10, 58.8%		

### Proteomic Analysis of CSF of Patients With MS and NINCs

**Figure 1:**
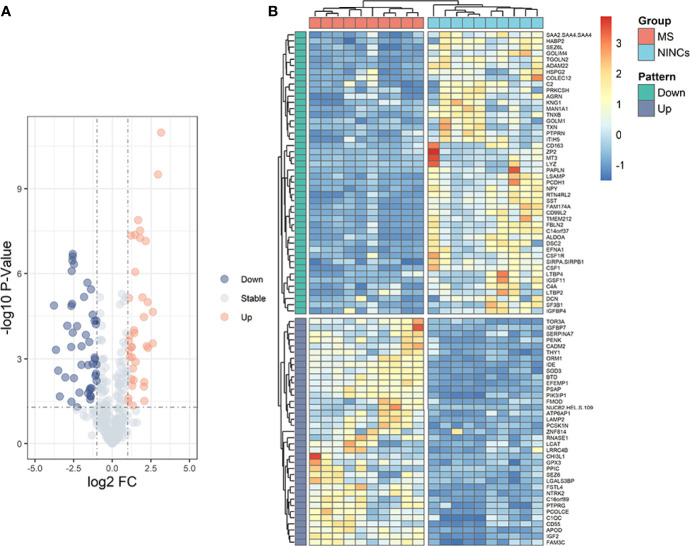
Volcano plot **(A)** and heatmap **(B)** of patients with MS *vs* NINCs. **(A)** The volcano plot was drawn using two factors, the fold change (Log2) between the two groups of samples and the p-value (−Log10) obtained from the t-test, to show the significance of differences in the data between the two groups of samples. The orange dots and blue dots in the figure are proteins that were significantly upregulated and downregulated, respectively; the gray dots are proteins with no significant difference. **(B)** Hierarchical clustering analysis of DEPs in CSF between patients with MS (orange) and NINCs (blue). Each row in the figure represents a protein, and each column represents a sample. Red, high expression; dark blue, low expression. Two main clusters of proteins were observed, one of which was downregulated and one of which upregulated in patients with MS.

In the discovery cohort, 343 proteins were identified. Significantly differentially expressed proteins (DEPs) were screened for fold change ≥ 2 or ≤ 0.5, and *P*< 0.05 (**Supplementary Table S1**). We found 83 differentially expressed proteins (DEPs) in the MS group compared to NINCs (**Supplementary Table S2**). Of these DEPs, the expression level of 37 proteins (44.6%) increased and 46 proteins (55.4%) decreased in MS patients. The volcano plot describes the distribution of all the proteins based on *P* values and MS/NINCs abundance ratio (**Figure 1A**). The cluster analysis for DEPs expression clearly showed that the expression pattern of MS patients differed from NINCs, and that the protein expression in every group was clustered together. A heatmap of all 83 DEPs is presented in **Figure 1B**.

### Functional Categorization of Differentially Expressed Proteins in MS Patients

**Figure 2:**
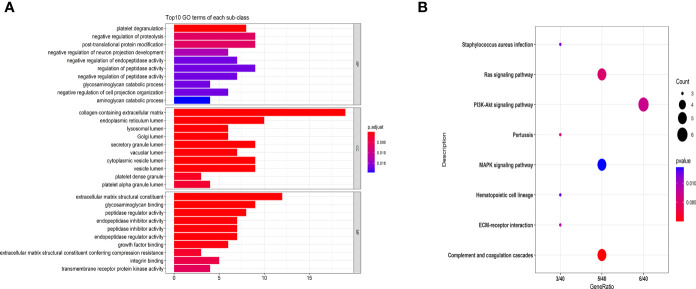
GO **(A)** and KEGG pathway analyses **(B)** of MS-related proteins. Classification of 83 DEPs based on biological processes, molecular functions, cellular components **(A)** and KEGG pathways **(B)**. **(A)** The abscissa represents the number of DEPs in each functional classification. **(B)** The ordinate represents the significantly enriched KEGG pathway, and the abscissa represents the number of genes enriched in the KEGG pathway/total number of genes. The colors in the figure indicate the magnitude of the p-value. The size of the point represents the gene number.

To better understand DEPs functions, we performed GO analysis. As indicated in **Figure 2A**, GO analysis results were considered statistically significant at *P*<0.05. At the level of biological process (BP), the significantly enriched GO terms included negative regulation of proteolysis, post-translational protein modification, regulation of peptidase activity, and platelet degranulation. The significantly enriched GO terms in the cellular component (CC) included collagen-containing extracellular matrix, endoplasmic reticulum lumen, and cytoplasmic vesicle lumen. The abundant GO terms in the molecular function (MF) included extracellular matrix structural constituents, glycosaminoglycan binding, and peptidase regulator activity.

Additionally, we performed the KEGG pathway analysis of DEPs, and the most significantly enriched pathways are shown in **Figure 2B**. The significantly regulated proteins were mainly involved in the PI3K-Akt signaling pathway, complement and coagulation cascades, Ras signaling pathway, and MAPK signaling pathway.

### Interaction Network Analysis of the DEPs

**Figure 3:**
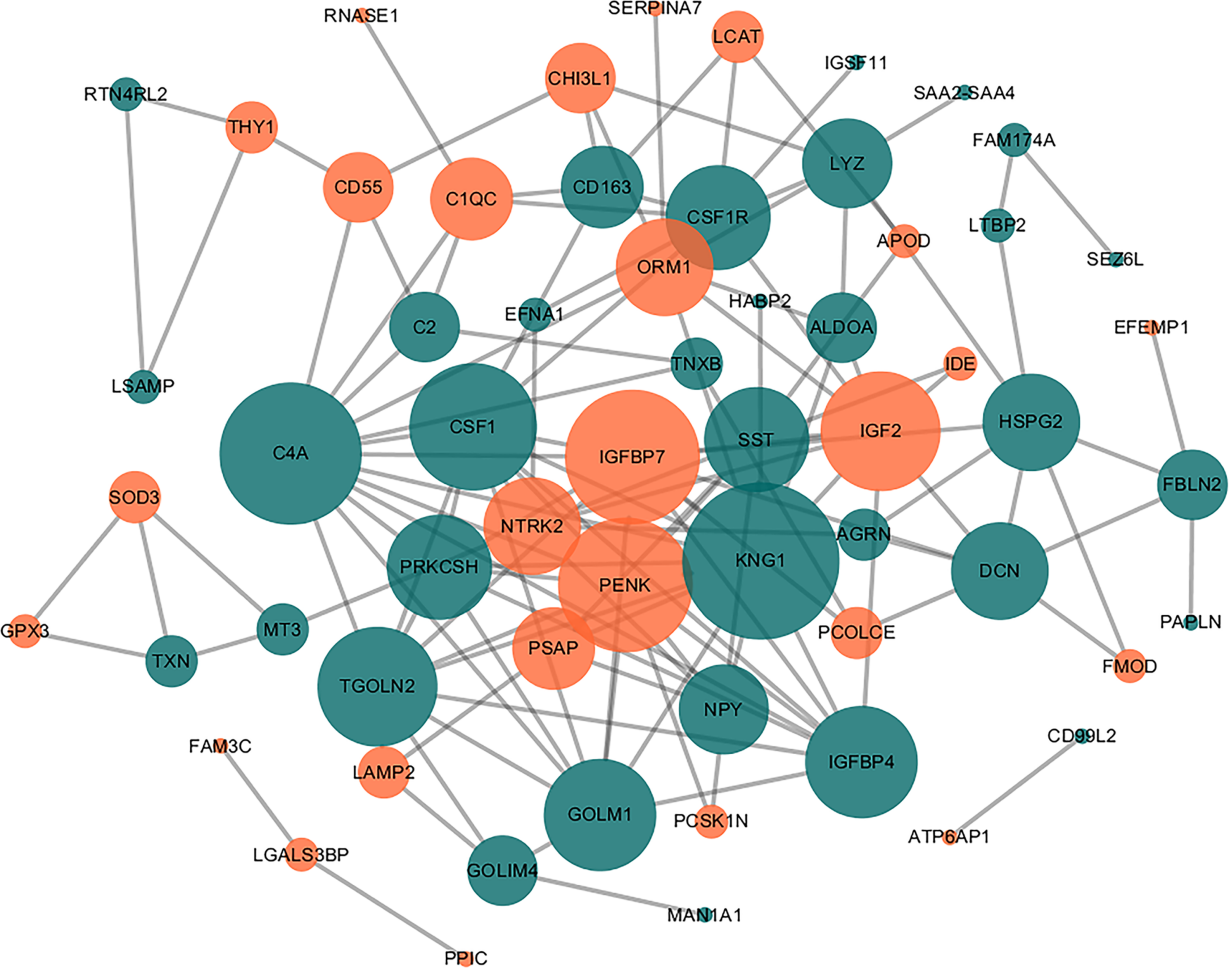
Direct PPI network. Sixty of the 83 DEPs were predicted to participate in direct PPIs, and the interactions were based on the ‘evidence’ mode and a moderate level of confidence. Nodes represent proteins and edges represent PPIs. The degree determines the node size, where orange represents upregulated and blue represents downregulated.

Using the STRING PPI database and Cytoscape tools, a PPI network was established and we found that 60 DEPs exhibited direct interactions, as shown in **Figure 3**. The nodes in the figure represented differentially expressed proteins, and the down-regulated proteins in green and up-regulated proteins in orange. The size of the node to reflect the degree of the node was set using the Cytoscape tool. In the network, the number of proteins that directly interact with a certain protein A is called the connection degree of the protein A. Generally speaking, the greater the connection degree of a protein, the greater the disturbance to the entire system when the protein changes, and this protein may be the key to maintaining the balance and stability of the system. We used the Cytoscape tool to set the size of the node to reflect the degree of the node. A larger node represents a higher degree of the node. After analyzing node degree using this network, the top 10 proteins in the degree of the nodes in this network include: KNG1, C4A, IGFBP7, PENK, CSF1, IGF2, TGOLN2, IGFBP4, GOLM1, SST. In these top 10 proteins, IGF2, IGBP7 and SST were the proteins with large fold changes (FC=4.43, 2.60 and 0.17).Besides, we found that IGF2, IGFBP7 and SST are related to demyelinating disease in previous research (19–21). As a result, we selected the three proteins as the putative candidate proteins for subsequent experimental validation, including IGFBP7, IGF2, and SST.

### Validation of DEPs using ELISA

**Figure 4:**
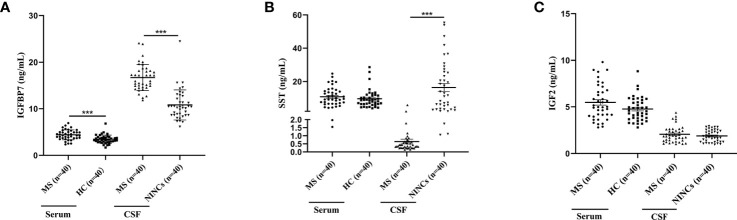
Dot Plots showing levels of IGFBP7, SST and IGF2 in serum and CSF samples of MS and controls. **(A)** IGFBP7 measurements in the serum and CSF of patients with MS and controls. **(B)** SST measurements in the serum and CSF of patients with MS and controls. **(C)** IGF2 measurements in the serum and CSF of patients with MS and controls. For simplicity, only significant differences are shown. Statistical significance was defined as ***P < 0.001.

The selected DEPs were validated using the commercial ELISA kit and both CSF and serum samples obtained from a different cohort. Compared to NINCs, the level of CSF IGFBP7 was significantly upregulated, and the level of CSF SST was significantly downregulated in the MS group (**Figures 4A, B**). This result is consistent with the results of our proteomics analysis. However, the expression level of IGF2 proteins did not change significantly, which is not consistent with our proteomic results (**Figure 4C**). Additionally, the serum levels of these three DEPs were also validated at the same time. Interestingly, compared to the control group, only the level of IGFBP7 in MS patients was increased in the serum, which is consistent with the result in CSF (**Figure 4A**). The other two proteins did not significantly change in the serum of MS patients (**Figures 4B, C**).

### Association of SST and IGFBP7 With MS Phenotypes

**Figure 5:**
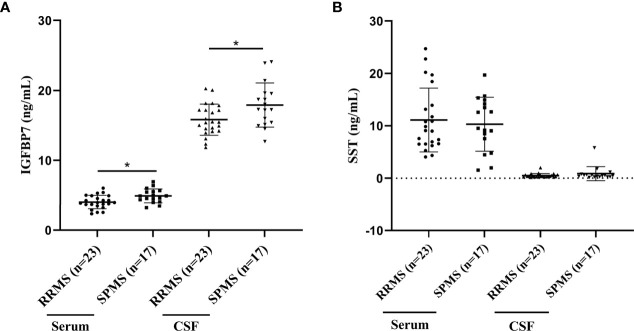
IGFBP7 and SST measurements in study subjects. **(A)** IGFBP7 measurements in the serum and CSF of patients with RRMS and SPMS. **(B)** SST measurements in the serum and CSF of patients with RRMS and SPMS. For simplicity, only significant differences are shown. Statistical significance was defined as *P < 0.05.

According to the 2013 update of the MS disease classification, the disease activity was introduced as marker of the two abundant MS phenotypes, RRMS and progressive MS (PPMS, SPMS) (22). In our validation cohort, 23 patients were diagnosed with RRMS, and 17 with SPMS. Upon comparing RRMS and SPMS patients, both the serum and CSF IGFBP7 levels were markedly elevated in SPMS patients compared to RRMS patients (*P*=0.011 and *P*=0.026, respectively; **Figure 5A**). However, both the serum and CSF SST levels were not significantly different between patients with SPMS and RRMS (*P*=0.914 and *P*=0.477, respectively; **Figure 5B**).

### Correlation Between Serum and CSF Measurements of IGFBP7

**Figure 6:**
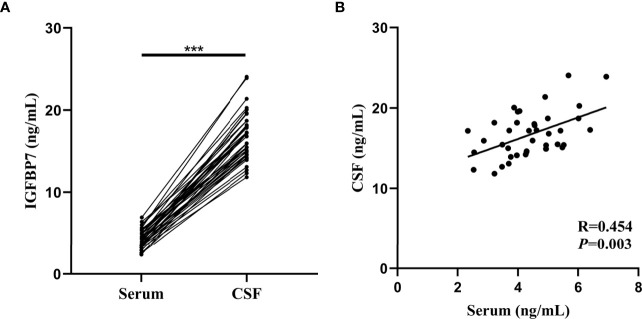
Comparison and correlation between IGFBP7 in the serum and matched CSF of MS patients. **(A)** IGFBP7 measurements in CSF and matched serum samples collected from 40 patients. **(B)** Correlation plots between CSF and serum IFGBP7 in individual MS patients (n=40). Pearson’s correlation coefficients (R) and P-values are shown. Statistical significance was defined as ***P < 0.001.

Both the serum and CSF IGFBP7 levels of all MS patients from the validation cohort were assessed. According to **Figure 6A**, the median concentration of CSF IGFBP7 was 16.395 (11.848-24.072) ng/mL, which was significantly higher than that in the serum (4.333 ng/mL, 2.351-6.933 ng/mL; *P*=0.002). Interestingly, serum IGFBP7 levels were positively correlated with CSF IGFBP7 levels in MS patients (R=0.454, *P*=0.003, **Figure 6B**).

### Evaluation of the Diagnostic Efficacy of SST and IGFBP7 in MS

**Figure 7:**
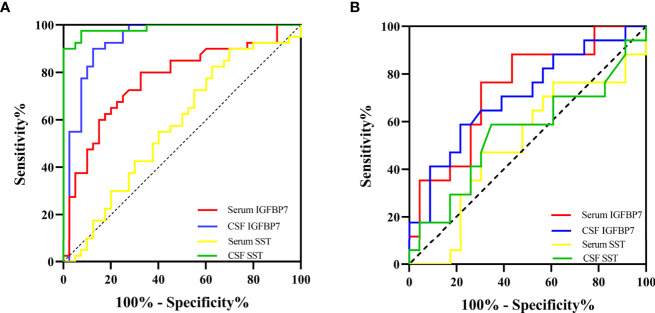
The diagnostic value of IGFBP7 and SST in MS. **(A)** ROC curvers for diagosing MS. **(B)** ROC curves for distinguishing SPMS from RRMS.

The area under the curve (AUC) generated by receiver operating characteristic (ROC) curve analysis indicated the diagnostic efficacy of both SST and IGFBP7 in MS patients, as shown in **Table 2**. The CSF levels of SST, IGFBP7, and the serum levels of IGFBP7 were demonstrated to exhibit notable efficacy in distinguishing MS from NINCs (**Figure 7A**). The most commonly used optimality criterion for cut-off point selection in the context of ROC curve analysis is the maximum of the Youden index. The Youden index, which is defined as the maximum of (sensitivity + specificity -1), directly measures the largest total diagnostic accuracy a biomarker can achieve. CSF IGFBP7 had a cut-off of 13.8 ng/mL with 90% sensitivity and 87.5% specificity, while serum IGFBP7 had a cut-off of 3.7 ng/mL with 80% sensitivity and 67.5% specificity (**Table 3**). Comparatively, the cut-off for CSF SST was 2.0 ng/mL with 97.5% sensitivity and 92.5% specificity (**Table 3**). These findings indicate that CSF IGFBP7, serum IGFBP7, and CSF SST are reliable biomarkers of MS.

**Table 2:** Diagnostic value of SST and IGFBP7 in MS.

Biomarkers	AUC (95% CI)	
	MS *vs*. control	RRMS *vs*. SPMS
CSF SST	0.986 (0.966-1.000)*	0.552 (0.363-0.742)
Serum SST	0.578 (0.451-0.704)	0.506 (0.320-0.693)
CSF IGFBP7	0.931 (0.871-0.992)*	0.707 (0.542-0.872)*
Serum IGFBP7	0.770 (0.664-0.876)*	0.747 (0.580-0.894)*

*P < 0.05.

**Table 3:** Biomarker characteristics.

Biomarkers	MS *vs*. control			RRMS *vs*. SPMS		
	Cut-off point* (ng/mL)	Sensitivity (%)	Specificity (%)	Cut-off point* (ng/mL)	Sensitivity (%)	Specificity (%)
CSF SST	2.0	97.5%	92.5%	0.4	58.8%	65.2%
Serum SST	12.3	37.5%	82.5%	12.1	47.1%	69.6%
CSF IGFBP7	13.8	90%	87.5%	17.2	58.8%	78.3%
Serum IGFBP7	3.7	80%	67.5%	4.3	76.5%	69.6%

*Cut-off point=maximum of (sensitivity + specificity -1).

Moreover, we researched the diagnostic capability of biomarkers for predicting SPMS. As demonstrated in **Table 2** and **Figure 7B**, both serum and CSF IGFBP7 exhibited remarkable predictive value for SPMS. The concern is that serum IGFBP7 had a higher accuracy in distinguishing SPMS from RRMS than CSF IGFBP7. Serum IGFBP7 had a cut-off of 4.3 ng/mL with 76.5% sensitivity and 69.6% specificity, whereas CSF IGFBP7 had a cut-off of 17.2ng/mL with 58.8% sensitivity and 78.3% specificity (**Table 3**). These findings indicate that both serum and CSF IGFBP7 can be used to predict the progression of MS.

## Discussion

Previously, it was difficult to discover reliable biomarkers for MS because of the clinical and pathophysiological complexities associated with this disease (23). Although approximately 10 to 15% of MS patients undergo a progressive course starting from the onset of the disease, which is known as primary progressive MS (PPMS), the disease progresses from an initial phase of RRMS to a subsequent phase of SPMS (24). The transition from RRMS to SPMS is a phenotypically gradual process, and thus, the diagnosis of SPMS by the clinicians is usually delayed leading to a clear sustained accumulation of disability. According to one study, the mean period of RRMS that turned to SPMS diagnostic uncertainty was 2.9 years (25). Therefore, there is an urgent need to identify molecular biomarkers to diagnose RRMS and monitor the disease progression to confirm SPMS.

In our study, we used TMT-based quantitative proteomics technology to analyze CSF of MS patients and NINCs to identify the potential biomarkers that can be used to diagnose MS. Strict exclusion criteria were set for the study subjects; using the 2-fold cutoff, we found that the expression of 37 proteins in MS patients was increased compared to NINCs, and that of 46 was decreased. Bioinformatics tools were used to analyze these proteins further, suggesting that these DEPs may play essential roles in the pathogenesis of MS. We finally chose three candidate biomarkers, IGFBP7, IGF2, and SST, for further analysis. We validated the three candidate proteins using an additional larger sample number as a validation cohort, and finally proved that the two proteins IGFBP7 and SST were significantly regulated in CSF of MS patients, which is consistent with our proteomic results in the discovery cohort (**Figure 4**). Surprisingly, the enhanced level of serum IGFBP7 was reproducibly observed in MS patients. Furthermore, CSF IGFBP7 was dramatically increased compared with that in the corresponding serum samples (**Figure 6A**). Subsequently, we found serum IGFBP7 levels were positively correlated with CSF IGFBP7 levels (R=0.454) (**Figure 6B**).

Next, we investigated the roles of candidate proteins in both CSF and serum for the diagnosis of MS and prediction of disease progression in MS. For MS diagnosis, the AUC obtained from the ROC curve was 0.986 (0.966–1.000) for CSF SST, which was higher than that for the CSF IGFBP7 [0.931 (0.871–0.992)] and serum IGFBP7 [0.770 (0.664–0.876)] (**Figure 7A** and **Table 2**). Accordingly, CSF SST and IGFBP7 exhibited good diagnostic performance for MS. The diagnostic ability was further evaluated to distinguish SPMS from RRMS, as serum IGFBP7 had a higher AUC (0.747) than CSF IGFBP7 (AUC: 0.707) (**Figure 7B**), which indicates that serum IGFBP7 may be a more appropriate marker as MS phenotypes than CSF IGFBP7 in clinical practice. In general, these results highlight the potential role of IGFBP7 in MS diagnosis and stratification.

Previous studies have revealed the presence of IGFBP in the choroid plexus of the central nervous system, in which IGFBP7 is secreted by astrocytes, followed by oligodendrocytes and neurons (26). IGFBP7 is a cell adhesive glycoprotein of approximately 30 kDa, which is a member of the IGFBP superfamily that modulates cell functions through IGF-dependent and -independent mechanisms (27). IGFBP7 is also known as IGFBP7-related protein 1 (IGFBP-rP1) or tumor-derived adhesion factor/angiomodulin (28). Increased IGFBP7 levels were found in CNS pathological conditions, such as glioblastoma and stroke, as well as EAE in a previous study (20, 29, 30). In our study, we found that IGFBP7 is upregulated in both CSF and serum of MS patients. In addition, serum IGFBP7 was positively correlated with CSF IGFBP7 (**Figure 6**) which suggested us IGFBP7 can be used to replace CSF test with serum test to reduce trauma. Our results, together with previous findings, support the hypothesis that IGFBP7 expression levels might be correlated with the degree of inflammatory demyelination. The remarkably high level of IGFBP7 in SPMS patients than in RRMS patients might be due to an increase in IGFBP7 expression as the disease progresses with an increased burden of inflammatory response. The role of IGFBP7 in the pathogenesis of MS remains largely unknown, and according to the GO analysis of IGFBP7 in MS, we suspected the possible pathogenesis involves post-translational protein modification. However, the specific pathogenesis mechanism needs to be further verified in subsequent studies.

The expression of IGF2 genes has been reported to be increased in inactive demyelinated lesions (19). IGF2 protein levels were also found to be increased in our study cohort. Therefore, we conducted a follow-up verification experiment using ELISA. However, we obtained inconsistent results, with no significant difference in IGF2 protein levels in the CSF or serum samples between the MS and control groups. However, further validation is required to conclude the results of this study.

Somatostatin (SST), a growth hormone inhibitory peptide, was found to play an important role in many CNS pathological conditions, such as Alzheimer’s disease, Parkinson’s disease, Huntington’s disease, and demyelinating disease (21, 31, 32). In recent years, many studies have been conducted to understand the role of SST in CNS diseases. Decrease of SST in CSF has been reported, which is associated with diminished cognitive function in MS patients. The low levels of SST in CSF during relapse in patients with MS were thought to be due to reduced or passive secretion of SST secreting cells (33). Consistent with these studies, we discovered a low expression of SST in CSF of MS patients in comparison to NINCs. However, similar results were not obtained for the serum samples.

Our study has some limitations, including that only CSF proteins of MS patients were screened, while the corresponding serum samples were not assessed for the proteomics analysis of the discovery cohort, and it was unknown whether IGFBP7 and SST levels were affected by therapy. Another limitation is our study excluded patients with cancer and/or cardiac diseases, whereas IGFBP7 expression was also found to be changed in these patients (26, 34). This should be taken into consideration in clinical application. Additionally, further research is needed to determine whether IGFBP7 is correlated with the other two forms of MS (PPMS and CIS). The current study is a preliminary exploration of the differentially expressed proteins in MS. More samples are needed to verify these conclusions to elucidate the underlying mechanisms of MS in the future.

## Conclusion

Conclusively, TMT-based proteomics technology provides a novel method for the identification of proteins involved in MS. Finally, our observations showed the significant value of SST and IGFBP7 for efficiently diagnosing and predicting the developmental phenotypes of MS, thereby providing a rationale for conducting further studies on the involvement of SST and IGFBP7 in the pathogenesis of MS.

## Data Availability Statement

The datasets presented in this study can be found in online repositories. The names of the repository/repositories and accession number(s) can be found below: http://www.proteomexchange.org/, IPX0002604000, PXD023027.

## Ethics Statement

The studies involving human participants were reviewed and approved by ethical committee of the Tiantan Hospital of Capital Medical University. The patients/participants provided their written informed consent to participate in this study. Written informed consent was obtained from the individual(s) for the publication of any potentially identifiable images or data included in this article.

## Author Contributions

GJZ and GHZ designed the study. YS and YD performed the research and drafted the manuscript. GL, LW, RO, JS and LQ participated in retrieving the literature. All authors contributed to the article and approved the submitted version.

## Funding

This study was supported by the Beijing Hospital Authority Clinical Medicine Development of Special Funding (grant no. ZYLX202108).

## Conflict of Interest

The authors declare that the research was conducted in the absence of any commercial or financial relationships that could be construed as a potential conflict of interest.

## Publisher’s Note

All claims expressed in this article are solely those of the authors and do not necessarily represent those of their affiliated organizations, or those of the publisher, the editors and the reviewers. Any product that may be evaluated in this article, or claim that may be made by its manufacturer, is not guaranteed or endorsed by the publisher.
