# Supplementary material for: Discovery of Novel Biomarkers for Diagnosing and Predicting the Progression of Multiple Sclerosis Using TMT-Based Quantitative Proteomics
Source: Front Immunol. 2021 Aug 20;12:700031. doi: 10.3389/fimmu.2021.700031 (PMC8417809; doi:10.3389/fimmu.2021.700031)
Supplement: Supplementary file 1 [file Table_1.docx]

**Supplementary tables**

Table S1 List of proteins identified in the CSF from MS patients compared with the NINCs.

| Protein ID | Protein names | MS/NINCs Ratios | Corrected FDR |
| --- | --- | --- | --- |
| P14735 | Insulin-degrading enzyme | 8.834 | 0.000 |
| P07602 | Prosaposin | 7.700 | 0.000 |
| Q6UX73 | UPF0764 protein C16orf89 | 6.123 | 0.000 |
| Q12841 | Follistatin-related protein 4 | 6.095 | 0.000 |
| P01210 | Proenkephalin-A | 4.927 | 0.000 |
| P13473 | Lysosome-associated membrane glycoprotein 2 | 4.857 | 0.000 |
| Q8N3J6 | Cell adhesion molecule 2 | 4.642 | 0.000 |
| P01344 | Insulin-like growth factor II | 4.431 | 0.000 |
| P45877 | Peptidyl-prolyl cis-trans isomerase C | 4.172 | 0.030 |
| Q06828 | Fibromodulin | 4.133 | 0.006 |
| P36222 | Chitinase-3-like protein 1 | 4.096 | 0.009 |
| Q5W0C9 | Torsin-3A | 4.092 | 0.000 |
| P02763 | Alpha-1-acid glycoprotein 1 | 3.830 | 0.000 |
| Q16620 | BDNF/NT-3 growth factors receptor | 3.463 | 0.000 |
| P43251 | Biotinidase | 3.450 | 0.000 |
| Q580Q6 | EGF-containing fibulin-like extracellular matrix protein 1 | 3.156 | 0.000 |
| P12111 | Collagen alpha-3(VI) chain | 3.015 | 0.182 |
| O94772 | Lymphocyte antigen 6H | 2.917 | 0.070 |
| P07998 | Ribonuclease pancreatic | 2.820 | 0.001 |
| Q96FE7 | Phosphoinositide-3-kinase-interacting protein 1 | 2.771 | 0.000 |
| P08294 | Extracellular superoxide dismutase [Cu-Zn] | 2.742 | 0.000 |
| Q15113 | Procollagen C-endopeptidase enhancer 1 | 2.674 | 0.000 |
| P04216 | Thy-1 membrane glycoprotein | 2.641 | 0.000 |
| Q16270 | Insulin-like growth factor-binding protein 7 | 2.599 | 0.006 |
| P02656 | Apolipoprotein C-III | 2.590 | 0.182 |
| Q53EL9 | Seizure protein 6 homolog | 2.513 | 0.002 |
| P04180 | Phosphatidylcholine-sterol acyltransferase | 2.507 | 0.044 |
| P80303 | Nucleobindin-2 | 2.447 | 0.009 |
| P02747 | Complement C1q subcomponent subunit C | 2.412 | 0.001 |
| P08174 | Complement decay-accelerating factor | 2.399 | 0.000 |
| P22352 | Glutathione peroxidase;Glutathione peroxidase 3 | 2.342 | 0.000 |
| Q15904 | V-type proton ATPase subunit S1 | 2.336 | 0.005 |
| P05090 | Apolipoprotein D | 2.297 | 0.000 |
| P23470 | Protein-tyrosine-phosphatase;Receptor-type tyrosine-protein phosphatase gamma | 2.174 | 0.000 |
| Q08380 | Galectin-3-binding protein | 2.171 | 0.001 |
| P05543 | Thyroxine-binding globulin | 2.108 | 0.000 |
| Q92520 | Protein FAM3C | 2.106 | 0.000 |
| Q9UHG2 | ProSAAS;KEP;Big SAAS;Little SAAS;Big PEN-LEN;PEN;Little LEN;Big LEN | 2.097 | 0.000 |
| M0R066 | Putative uncharacterized zinc finger protein 814 | 2.044 | 0.024 |
| Q9NT99 | Leucine-rich repeat-containing protein 4B | 2.005 | 0.031 |
| O94985 | Carbohydrate sulfotransferase 15 | 1.960 | 0.101 |
| P19652 | Alpha-1-acid glycoprotein 2 | 1.947 | 0.003 |
| Q8IUX7 | Adipocyte enhancer-binding protein 1 | 1.916 | 0.371 |
| P68871 | Hemoglobin subunit beta;LVV-hemorphin-7;Spinorphin | 1.912 | 0.466 |
| P78509 | Reelin | 1.873 | 0.000 |
| P00748 | Coagulation factor XII | 1.849 | 0.002 |
| Q6P672 | Complement C1r subcomponent-like protein | 1.848 | 0.003 |
| P29120 | Neuroendocrine convertase 1 | 1.845 | 0.157 |
| P11362 | Fibroblast growth factor receptor | 1.837 | 0.004 |
| P08603 | Complement factor H | 1.825 | 0.000 |
| P01871 | Immunoglobulin heavy constant mu | 1.787 | 0.007 |
| P00441 | Superoxide dismutase [Cu-Zn] | 1.780 | 0.000 |
| P99999 | Cytochrome c | 1.776 | 0.008 |
| O75882 | Attractin | 1.705 | 0.147 |
| P41222 | Prostaglandin-H2 D-isomerase | 1.671 | 0.000 |
| P24043 | Laminin subunit alpha-2 | 1.667 | 0.078 |
| O75711 | Scrapie-responsive protein 1 | 1.664 | 0.124 |
| Q99435 | Protein kinase C-binding protein NELL2 | 1.624 | 0.000 |
| Q99983 | Osteomodulin | 1.623 | 0.012 |
| Q92820 | Gamma-glutamyl hydrolase | 1.614 | 0.029 |
| P28290 | Sperm-specific antigen 2 | 1.612 | 0.127 |
| P07195 | L-lactate dehydrogenase B chain;L-lactate dehydrogenase | 1.593 | 0.057 |
| P34096 | Ribonuclease 4 | 1.592 | 0.615 |
| P01034 | Cystatin-C | 1.587 | 0.003 |
| P48740 | Mannan-binding lectin serine protease 1 | 1.583 | 0.304 |
| P06865 | Beta-hexosaminidase;Beta-hexosaminidase subunit alpha | 1.573 | 0.318 |
| P51884 | Lumican | 1.553 | 0.001 |
| P30530 | Tyrosine-protein kinase receptor UFO | 1.533 | 0.109 |
| P02760 | Protein AMBP | 1.530 | 0.000 |
| Q92563 | Testican-2 | 1.525 | 0.212 |
| Q9BY67 | Cell adhesion molecule 1 | 1.521 | 0.138 |
| Q9NQ79 | Cartilage acidic protein 1 | 1.514 | 0.169 |
| Q5SPY9 | Neural proliferation differentiation and control protein 1 | 1.498 | 0.016 |
| P02461 | Collagen alpha-1(III) chain | 1.494 | 0.221 |
| P20774 | Mimecan | 1.490 | 0.005 |
| Q6U2M2 | Complement C4-B | 1.480 | 0.001 |
| Q12907 | Vesicular integral-membrane protein VIP36 | 1.476 | 0.155 |
| P08670 | Vimentin | 1.474 | 0.255 |
| P01042 | Kininogen-1 | 1.464 | 0.002 |
| O94985 | Calsyntenin-1 | 1.455 | 0.140 |
| O43505 | Beta-1,4-glucuronyltransferase 1 | 1.445 | 0.027 |
| P12259 | Coagulation factor V | 1.443 | 0.179 |
| A3KFI4 | Neuroblastoma suppressor of tumorigenicity 1 | 1.432 | 0.336 |
| P23284 | Peptidyl-prolyl cis-trans isomerase B | 1.420 | 0.035 |
| Q9Y4C0 | Neurexin-3 | 1.410 | 0.080 |
| O14498 | Immunoglobulin superfamily containing leucine-rich repeat protein | 1.405 | 0.221 |
| Q9UBX5 | Fibulin-5 | 1.405 | 0.000 |
| P13521 | Secretogranin-2 | 1.404 | 0.007 |
| Q24JP5 | Transmembrane protein 132A | 1.401 | 0.567 |
| P04196 | Histidine-rich glycoprotein | 1.389 | 0.003 |
| P01023 | Alpha-2-macroglobulin | 1.380 | 0.008 |
| P02749 | Beta-2-glycoprotein 1 | 1.378 | 0.155 |
| Q6IN67 | Hypoxia up-regulated protein 1 | 1.377 | 0.001 |
| O00584 | Ribonuclease T2 | 1.377 | 0.060 |
| O75503 | Ceroid-lipofuscinosis neuronal protein 5 | 1.367 | 0.167 |
| P02765 | Alpha-2-HS-glycoprotein | 1.367 | 0.059 |
| P54289 | Voltage-dependent calcium channel subunit alpha-2/delta-1 channel subunit delta-1 | 1.345 | 0.015 |
| P00746 | Complement factor D | 1.325 | 0.524 |
| P17174 | Aspartate aminotransferase, cytoplasmic | 1.323 | 0.408 |
| P55290 | Cadherin-13 | 1.317 | 0.009 |
| P35858 | Insulin-like growth factor-binding protein complex acid labile subunit | 1.317 | 0.628 |
| Q14118 | Dystroglycan | 1.313 | 0.008 |
| P02654 | Apolipoprotein C-I | 1.299 | 0.442 |
| Q96PD5 | N-acetylmuramoyl-L-alanine amidase | 1.295 | 0.194 |
| P19021 | Peptidyl-glycine alpha-amidating monooxygenase | 1.286 | 0.239 |
| Q8WVQ1 | Soluble calcium-activated nucleotidase 1 | 1.286 | 0.183 |
| Q9BRK5 | 45 kDa calcium-binding protein | 1.284 | 0.134 |
| O94919 | Endonuclease domain-containing 1 protein | 1.283 | 0.387 |
| P10909 | Clusterin | 1.281 | 0.206 |
| O00533 | Neural cell adhesion molecule L1-like protein | 1.276 | 0.022 |
| P05155 | Plasma protease C1 inhibitor | 1.271 | 0.005 |
| P61916 | Epididymal secretory protein E1 | 1.261 | 0.124 |
| Q02246 | Contactin-2 | 1.259 | 0.136 |
| P09871 | Complement C1s subcomponent | 1.248 | 0.054 |
| P02790 | Hemopexin | 1.248 | 0.056 |
| P10643 | Complement component C7 | 1.240 | 0.139 |
| P01009 | Protein Z-dependent protease inhibitor | 1.238 | 0.634 |
| O94856 | Neurofascin | 1.232 | 0.462 |
| P07858 | Cathepsin B | 1.232 | 0.339 |
| P07225 | Vitamin K-dependent protein S | 1.228 | 0.287 |
| O43405 | Cochlin | 1.228 | 0.615 |
| P00915 | Carbonic anhydrase 1 | 1.225 | 0.716 |
| P16035 | Metalloproteinase inhibitor 2 | 1.210 | 0.227 |
| H7C1H0 | Insulin-like growth factor-binding protein 2 | 1.208 | 0.020 |
| Q92859 | Neogenin | 1.205 | 0.328 |
| P21802 | Fibroblast growth factor receptor | 1.194 | 0.656 |
| P40189 | Interleukin-6 receptor subunit beta | 1.192 | 0.328 |
| P22792 | Carboxypeptidase N subunit 2 | 1.171 | 0.507 |
| P32119 | Peroxiredoxin-2 | 1.169 | 0.794 |
| Q03591 | Complement factor H-related protein 1 | 1.168 | 0.599 |
| P23142 | Fibulin-1 | 1.168 | 0.004 |
| P43652 | Afamin | 1.166 | 0.134 |
| O95445 | Apolipoprotein M | 1.165 | 0.461 |
| Q13822 | Ectonucleotide pyrophosphatase | 1.163 | 0.170 |
| P62158 | Brevican core protein | 1.158 | 0.396 |
| P07360 | Complement component C8 gamma chain | 1.150 | 0.585 |
| Q53YY1 | Angiotensinogen | 1.147 | 0.161 |
| P04217 | Alpha-1B-glycoprotein | 1.146 | 0.219 |
| P01008 | Antithrombin-III | 1.140 | 0.272 |
| O95196 | Chondroitin sulfate proteoglycan 5 | 1.139 | 0.698 |
| P61769 | Beta-2-microglobulin | 1.132 | 0.461 |
| P00747 | Plasminogen | 1.124 | 0.329 |
| Q92823 | Neuronal cell adhesion molecule | 1.119 | 0.164 |
| P55058 | Phospholipid transfer protein | 1.118 | 0.589 |
| P05156 | Complement factor I | 1.116 | 0.453 |
| P09972 | Fructose-bisphosphate aldolase C;Fructose-bisphosphate aldolase | 1.114 | 0.614 |
| P08697 | Alpha-2-antiplasmin | 1.112 | 0.291 |
| P14618 | Pyruvate kinase PKM | 1.103 | 0.582 |
| P60174 | Triosephosphate isomerase | 1.095 | 0.605 |
| Q92876 | Kallikrein-6 | 1.090 | 0.460 |
| Q15582 | Transforming growth factor-beta-induced protein ig-h3 | 1.087 | 0.536 |
| O60888 | Protein CutA | 1.086 | 0.823 |
| P08253 | 72 kDa type IV collagenase;PEX | 1.085 | 0.454 |
| Q14515 | SPARC | 1.081 | 0.755 |
| Q7Z3B1 | Neuronal growth regulator 1 | 1.080 | 0.744 |
| Q5J875 | Fetuin-B | 1.072 | 0.725 |
| P04004 | Vitronectin | 1.070 | 0.592 |
| P36955 | Pigment epithelium-derived factor | 1.068 | 0.679 |
| P69905 | Hemoglobin subunit alpha | 1.063 | 0.917 |
| P04156 | Major prion protein | 1.062 | 0.688 |
| O43556 | Epsilon-sarcoglycan | 1.060 | 0.874 |
| P01009 | Alpha-1-antitrypsin | 1.056 | 0.616 |
| P02649 | Apolipoprotein E | 1.048 | 0.727 |
| O75093 | Slit homolog 1 protein | 1.046 | 0.839 |
| P43121 | Cell surface glycoprotein MUC18 | 1.043 | 0.848 |
| Q96KN2 | Beta-Ala-His dipeptidase | 1.042 | 0.635 |
| Q8N126 | Cell adhesion molecule 3 | 1.033 | 0.782 |
| Q02818 | Nucleobindin-1 | 1.032 | 0.883 |
| P02746 | Complement C1q subcomponent subunit B | 1.032 | 0.799 |
| P17900 | Ganglioside GM2 activator | 1.024 | 0.923 |
| P00450 | Ceruloplasmin | 1.024 | 0.844 |
| Q9Y646 | Carboxypeptidase Q | 1.022 | 0.860 |
| P29622 | Kallistatin | 1.017 | 0.909 |
| Q969P0 | Immunoglobulin superfamily member 8 | 1.015 | 0.939 |
| P58401 | Neurexin-2 | 1.014 | 0.916 |
| P07108 | Acyl-CoA-binding protein | 1.010 | 0.960 |
| Q15818 | Neuronal pentraxin-1 | 1.009 | 0.957 |
| P02753 | Retinol-binding protein 4 | 0.999 | 0.996 |
| Q14515 | SPARC-like protein 1 | 0.994 | 0.969 |
| Q16610 | Extracellular matrix protein 1 | 0.993 | 0.966 |
| P02774 |  | 0.991 | 0.924 |
| P06727 | Apolipoprotein A-IV | 0.990 | 0.933 |
| P13987 | CD59 glycoprotein | 0.983 | 0.942 |
| P01011 | Alpha-1-antichymotrypsin | 0.975 | 0.836 |
| P10451 | Osteopontin | 0.973 | 0.856 |
| P07358 | Complement component C8 beta chain | 0.972 | 0.933 |
| P19022 | Cadherin-2 | 0.969 | 0.767 |
| P05546 | Heparin cofactor 2 | 0.968 | 0.829 |
| Q6ZSJ9 | Protein shisa-6 homolog | 0.968 | 0.923 |
| P63267 | Actin, cytoplasmic 2 | 0.967 | 0.811 |
| P13611 | Versican core protein | 0.960 | 0.845 |
| Q8NFZ8 | Cell adhesion molecule 4 | 0.956 | 0.761 |
| P07339 | Cathepsin D | 0.948 | 0.670 |
| P00734 | Prothrombin | 0.947 | 0.693 |
| P52797 | Ephrin-A3 | 0.935 | 0.810 |
| P02452 | Collagen alpha-1(I) chain | 0.923 | 0.578 |
| Q6UXB8 | Peptidase inhibitor 16 | 0.917 | 0.731 |
| Q99969 | Retinoic acid receptor responder protein 2 | 0.912 | 0.816 |
| P00736 | Complement C1r subcomponent | 0.911 | 0.370 |
| P08571 | Monocyte differentiation antigen CD14 | 0.904 | 0.446 |
| Q6PQ81 | Dickkopf-related protein 3 | 0.897 | 0.369 |
| Q6UX71 | Podocalyxin-like protein 2 | 0.895 | 0.802 |
| P15259 | Phosphoglycerate mutase 2 | 0.894 | 0.693 |
| Q12860 | Contactin-1 | 0.892 | 0.110 |
| Q99674 | Cell growth regulator with EF hand domain protein 1 | 0.844 | 0.700 |
| P02751 | Fibronectin | 0.842 | 0.072 |
| P18428 | Lipopolysaccharide-binding protein | 0.836 | 0.675 |
| P13591 | Neural cell adhesion molecule 1 | 0.836 | 0.027 |
| P35908 | Keratin, type II cytoskeletal 2 epidermal | 0.834 | 0.539 |
| P06396 | Gelsolin | 0.829 | 0.044 |
| P0CG04 | Immunoglobulin lambda-like polypeptide 5 | 0.825 | 0.168 |
| P05060 | Secretogranin-1 | 0.809 | 0.203 |
| P05452 | Tetranectin | 0.800 | 0.278 |
| P07357 | Complement component C8 alpha chain | 0.792 | 0.110 |
| P23435 | Cerebellin-1 | 0.789 | 0.153 |
| P16070 | CD44 antigen | 0.788 | 0.006 |
| P25311 | Zinc-alpha-2-glycoprotein | 0.788 | 0.093 |
| Q05BT0 | Multiple epidermal growth factor-like domains protein 8 | 0.788 | 0.303 |
| P40925 | Malate dehydrogenase, cytoplasmic | 0.788 | 0.634 |
| P05408 | Neuroendocrine protein 7B2 | 0.787 | 0.419 |
| P01033 | Metalloproteinase inhibitor 1 | 0.782 | 0.080 |
| P19823 | Inter-alpha-trypsin inhibitor heavy chain H2 | 0.781 | 0.091 |
| P12109 | Collagen alpha-1(VI) chain | 0.773 | 0.042 |
| P23142 | Fibulin-1 | 0.768 | 0.031 |
| P30086 | Phosphatidylethanolamine-binding protein 1 | 0.766 | 0.251 |
| P32119 | Peroxiredoxin-1 | 0.765 | 0.492 |
| P10645 | Chromogranin-A | 0.759 | 0.060 |
| P27169 | Serum paraoxonase/arylesterase 1 | 0.757 | 0.084 |
| Q12841 | Follistatin-related protein 1 | 0.755 | 0.048 |
| P07711 | Cathepsin L1 | 0.754 | 0.262 |
| Q96PX8 | SLIT and NTRK-like protein 1 | 0.747 | 0.528 |
| Q8WXD2 | Secretogranin-3 | 0.742 | 0.002 |
| Q13740 | CD166 antigen | 0.740 | 0.221 |
| P05067 | Amyloid beta A4 protein | 0.740 | 0.040 |
| Q9BTY2 | Plasma alpha-L-fucosidase | 0.738 | 0.400 |
| P16870 | Carboxypeptidase E | 0.738 | 0.156 |
| P23471 | Receptor-type tyrosine-protein phosphatase zeta | 0.730 | 0.013 |
| P62937 | Peptidyl-prolyl cis-trans isomerase | 0.726 | 0.282 |
| O15240 | Neurosecretory protein VGF | 0.719 | 0.068 |
| Q96S96 | Phosphatidylethanolamine-binding protein 4 | 0.710 | 0.075 |
| P02748 | Complement component C9 | 0.703 | 0.041 |
| Q06481 | Amyloid-like protein 2 | 0.699 | 0.197 |
| P04003 | C4b-binding protein alpha chain | 0.693 | 0.374 |
| P13671 | Complement component C6 | 0.692 | 0.237 |
| P00751 | Complement factor B | 0.691 | 0.014 |
| P58400 | Neurexin-1 | 0.687 | 0.051 |
| Q14624 | Inter-alpha-trypsin inhibitor heavy chain H4 | 0.686 | 0.006 |
| P32004 | Neural cell adhesion molecule L1 | 0.683 | 0.151 |
| Q9HC57 | WAP four-disulfide core domain protein 1 | 0.680 | 0.358 |
| Q99574 | Neuroserpin | 0.679 | 0.180 |
| P27797 | Calreticulin | 0.679 | 0.058 |
| P51693 | Amyloid-like protein 1;C30 | 0.677 | 0.001 |
| P35527 | Keratin, type I cytoskeletal 9 | 0.661 | 0.128 |
| P01031 | Complement C5 | 0.652 | 0.047 |
| Q6UX71 | Plexin domain-containing protein 2 | 0.650 | 0.097 |
| O95502 | Neuronal pentraxin receptor | 0.647 | 0.005 |
| O15394 | Neural cell adhesion molecule 2 | 0.645 | 0.001 |
| Q13332 | Protein-tyrosine-phosphatase | 0.635 | 0.007 |
| P08123 | Collagen alpha-2(I) chain | 0.633 | 0.003 |
| P05155 | Selenoprotein P | 0.630 | 0.011 |
| Q6UXD5 | Seizure 6-like protein 2 | 0.627 | 0.016 |
| O14594 | Neurocan core protein | 0.625 | 0.000 |
| P08185 | Corticosteroid-binding globulin | 0.623 | 0.000 |
| P02750 | Leucine-rich alpha-2-glycoprotein | 0.619 | 0.001 |
| Q6UW01 | Cerebellin-3 | 0.615 | 0.157 |
| P14151 | L-selectin | 0.613 | 0.094 |
| O94769 | Extracellular matrix protein 2 | 0.603 | 0.112 |
| Q9P121 | Neurotrimin | 0.602 | 0.000 |
| Q8TAG5 | V-set and transmembrane domain-containing protein 2A | 0.598 | 0.094 |
| O60279 | Sushi domain-containing protein 5 | 0.586 | 0.033 |
| Q14982 | Opioid-binding protein/cell adhesion molecule | 0.586 | 0.010 |
| P19827 | Inter-alpha-trypsin inhibitor heavy chain H1 | 0.583 | 0.000 |
| P04114 | Apolipoprotein B-100 | 0.576 | 0.269 |
| O60883 | Prosaposin receptor GPR37L1 | 0.566 | 0.000 |
| P21246 | Pleiotrophin | 0.564 | 0.205 |
| P62328 | Thymosin beta-4 | 0.557 | 0.055 |
| Q8TEU8 | WAP four-disulfide core domain protein 1 | 0.552 | 0.005 |
| Q5T5P1 | Sickle tail protein homolog | 0.551 | 0.168 |
| Q86UD1 | Out at first protein homolog | 0.547 | 0.273 |
| P24592 | Insulin-like growth factor-binding protein 6 | 0.541 | 0.012 |
| C9JEM6 | Receptor protein-tyrosine kinase | 0.541 | 0.046 |
| P62987 | Ubiquitin-60S ribosomal protein L40 | 0.535 | 0.000 |
| P03952 | Plasma kallikrein | 0.532 | 0.010 |
| O75326 | Semaphorin-7A | 0.525 | 0.002 |
| P51693 | Amyloid-like protein 1;C30 | 0.524 | 0.010 |
| P62158 | Calmodulin | 0.509 | 0.207 |
| Q9Y5Y7 | Lymphatic vessel endothelial hyaluronic acid receptor 1 | 0.506 | 0.168 |
| P98160 | Basement membrane-specific heparan sulfate proteoglycan core protein | 0.493 | 0.006 |
| O00461 | Golgi membrane protein 1 | 0.491 | 0.002 |
| P78324 | Tyrosine-protein phosphatase non-receptor type substrate 1 | 0.487 | 0.000 |
| P01042 | Kininogen-1 | 0.478 | 0.003 |
| Q13449 | Limbic system-associated membrane protein | 0.475 | 0.000 |
| P09668 | Pro-cathepsin H | 0.469 | 0.121 |
| P06681 | Complement C2 | 0.468 | 0.004 |
| Q30211 | Complement C4-A | 0.459 | 0.001 |
| Q9NX62 | Inositol monophosphatase 3 | 0.454 | 0.065 |
| Q15952 | Agrin | 0.451 | 0.001 |
| Q14520 | Hyaluronan-binding protein 2 | 0.448 | 0.000 |
| P13645 | Keratin, type I cytoskeletal 10 | 0.432 | 0.079 |
| P13591 |  | 0.421 | 0.093 |
| P35542 | Serum amyloid A-4 protein | 0.415 | 0.000 |
| Q6ZSI9 | Calpain-12 | 0.413 | 0.061 |
| Q16849 | Receptor-type tyrosine-protein phosphatase-like N | 0.412 | 0.001 |
| P62258 | 14-3-3 protein epsilon | 0.399 | 0.509 |
| O75533 | Splicing factor 3B subunit 1 | 0.393 | 0.011 |
| P01303 | Pro-neuropeptide Y | 0.383 | 0.000 |
| Q14767 | Latent-transforming growth factor beta-binding protein 2 | 0.380 | 0.021 |
| Q5KU26 | Collectin-12 | 0.371 | 0.020 |
| P22692 | Insulin-like growth factor-binding protein 4 | 0.370 | 0.011 |
| Q86VB7 | Scavenger receptor cysteine-rich type 1 protein M130 | 0.369 | 0.026 |
| P07585 | Decorin | 0.361 | 0.012 |
| P98095 | Fibulin-2 | 0.354 | 0.000 |
| P14314 | Glucosidase 2 subunit beta | 0.337 | 0.002 |
| Q8N2S1 | Latent-transforming growth factor beta-binding protein 4 | 0.334 | 0.016 |
| Q9BYH1 | Seizure 6-like protein | 0.328 | 0.000 |
| P04264 | Keratin, type II cytoskeletal 1 | 0.317 | 0.116 |
| P20827 | Ephrin-A1;Ephrin-A1, secreted form | 0.304 | 0.000 |
| P04075 | Fructose-bisphosphate aldolase | 0.280 | 0.000 |
| Q01459 | Di-N-acetylchitobiase | 0.277 | 0.103 |
| P11047 | Laminin subunit gamma-1 | 0.271 | 0.063 |
| Q14624 | Inter-alpha-trypsin inhibitor heavy chain H5 | 0.251 | 0.000 |
| P07333 | Macrophage colony-stimulating factor 1 receptor | 0.249 | 0.002 |
| P25713 | Metallothionein | 0.229 | 0.015 |
| P61626 | Lysozyme | 0.205 | 0.050 |
| Q8TBP5 | Membrane protein FAM174A | 0.205 | 0.000 |
| P63104 | 14-3-3 protein zeta/delta | 0.202 | 0.065 |
| P33908 | Mannosyl-oligosaccharide 1,2-alpha-mannosidase IA | 0.179 | 0.005 |
| Q86UN3 | Reticulon-4 receptor-like 2 | 0.178 | 0.000 |
| O00461 | Golgi integral membrane protein 4 | 0.169 | 0.000 |
| P61278 | Somatostatin | 0.169 | 0.000 |
| P09603 | Macrophage colony-stimulating factor 1 | 0.168 | 0.000 |
| O43493 | Trans-Golgi network integral membrane protein 2 | 0.166 | 0.000 |
| Q86TY3 | Uncharacterized protein C14orf37 | 0.164 | 0.000 |
| P22105 | Tenascin-X | 0.161 | 0.000 |
| Q05996 | Zona pellucida sperm-binding protein 2 | 0.159 | 0.032 |
| Q08AL8 | Disintegrin and metalloproteinase domain-containing protein 22 | 0.159 | 0.000 |
| Q02487 | Desmocollin-2 | 0.158 | 0.000 |
| Q24JP5 | Transmembrane protein 212 | 0.133 | 0.000 |
| O95428 | Papilin | 0.122 | 0.004 |
| P10599 | Thioredoxin | 0.095 | 0.021 |
| Q5DX21 | Immunoglobulin superfamily member 11 | 0.088 | 0.003 |
| Q08174 | Protocadherin-1 | 0.080 | 0.000 |
| Q96E20 | CD99 antigen-like protein 2 | 0.074 | 0.000 |

Table S2 List of differentially expressed proteins identified in the CSF from MS patients compared with the NINCs.

| Protein ID | | Protein names | MS/NINCs Ratios | Corrected FDR |
| --- | --- | --- | --- | --- |
| P61626 | Lysozyme | | 0.205 | 0.049 |
| P04180 | Phosphatidylcholine-sterol acyltransferase | | 2.507 | 0.044 |
| Q05996 | Zona pellucida sperm-binding protein 2 | | 0.159 | 0.032 |
| Q9NT99 | Leucine-rich repeat-containing protein 4B | | 2.005 | 0.031 |
| P45877 | Peptidyl-prolyl cis-trans isomerase C | | 4.172 | 0.030 |
| Q86VB7 | Scavenger receptor cysteine-rich type 1 protein M130 | | 0.369 | 0.026 |
| M0R066 | Putative uncharacterized zinc finger protein 814 | | 2.044 | 0.024 |
| P10599 | Thioredoxin | | 0.095 | 0.021 |
| Q14767 | Latent-transforming growth factor beta-binding protein 2 | | 0.380 | 0.021 |
| Q5KU26 | Collectin-12 | | 0.371 | 0.020 |
| Q8N2S1 | Latent-transforming growth factor beta-binding protein 4 | | 0.334 | 0.016 |
| P25713 | Metallothionein | | 0.229 | 0.015 |
| P07585 | Decorin | | 0.361 | 0.012 |
| O75533 | Splicing factor 3B subunit 1 | | 0.393 | 0.011 |
| P22692 | Insulin-like growth factor-binding protein 4 | | 0.370 | 0.011 |
| P36222 | Chitinase-3-like protein 1 | | 4.096 | 0.009 |
| P80303 | Nucleobindin-2 | | 2.447 | 0.009 |
| Q06828 | Fibromodulin | | 4.133 | 0.006 |
| P98160 | Basement membrane-specific heparan sulfate proteoglycan core protein | | 0.493 | 0.006 |
| Q16270 | Insulin-like growth factor-binding protein 7 | | 2.599 | 0.006 |
| Q15904 | V-type proton ATPase subunit S1 | | 2.336 | 0.005 |
| P33908 | Mannosyl-oligosaccharide 1,2-alpha-mannosidase IA | | 0.179 | 0.005 |
| O95428 | Papilin | | 0.122 | 0.004 |
| P06681 | Complement C2 | | 0.468 | 0.004 |
| P01042 | Kininogen-1 | | 0.478 | 0.003 |
| Q5DX21 | Immunoglobulin superfamily member 11 | | 0.088 | 0.003 |
| Q53EL9 | Seizure protein 6 homolog | | 2.513 | 0.002 |
| P14314 | Glucosidase 2 subunit beta | | 0.337 | 0.002 |
| P07333 | Macrophage colony-stimulating factor 1 receptor | | 0.249 | 0.002 |
| O00461 | Golgi membrane protein 1 | | 0.491 | 0.002 |
| Q16849 | Receptor-type tyrosine-protein phosphatase-like N | | 0.412 | 0.001 |
| P07998 | Ribonuclease pancreatic | | 2.820 | 0.001 |
| Q30211 | Complement C4-A | | 0.459 | 0.001 |
| Q15952 | Agrin | | 0.451 | 0.001 |
| Q08380 | Galectin-3-binding protein | | 2.171 | 0.001 |
| P02747 | Complement C1q subcomponent subunit C | | 2.412 | 0.001 |
| Q08174 | Protocadherin-1 | | 0.080 | 0.000 |
| P05543 | Thyroxine-binding globulin | | 2.108 | 0.000 |
| P01210 | Proenkephalin-A | | 4.927 | 0.000 |
| P22352 | Glutathione peroxidase;Glutathione peroxidase 3 | | 2.342 | 0.000 |
| Q02487 | Desmocollin-2 | | 0.158 | 0.000 |
| P08174 | Complement decay-accelerating factor | | 2.399 | 0.000 |
| Q14624 | Inter-alpha-trypsin inhibitor heavy chain H5 | | 0.251 | 0.000 |
| Q8N3J6 | Cell adhesion molecule 2 | | 4.642 | 0.000 |
| Q6UX73 | UPF0764 protein C16orf89 | | 6.123 | 0.000 |
| P98095 | Fibulin-2 | | 0.354 | 0.000 |
| Q9UHG2 | ProSAAS;KEP;Big SAAS;Little SAAS;Big PEN-LEN;PEN;Little LEN;Big LEN | | 2.097 | 0.000 |
| P23470 | Protein-tyrosine-phosphatase;Receptor-type tyrosine-protein phosphatase gamma | | 2.174 | 0.000 |
| P20827 | Ephrin-A1;Ephrin-A1, secreted form | | 0.304 | 0.000 |
| Q15113 | Procollagen C-endopeptidase enhancer 1 | | 2.674 | 0.000 |
| O00461 | Golgi integral membrane protein 4 | | 0.169 | 0.000 |
| P04216 | Thy-1 membrane glycoprotein | | 2.641 | 0.000 |
| Q8TBP5 | Membrane protein FAM174A | | 0.205 | 0.000 |
| Q24JP5 | Transmembrane protein 212 | | 0.133 | 0.000 |
| Q13449 | Limbic system-associated membrane protein | | 0.475 | 0.000 |
| Q14520 | Hyaluronan-binding protein 2 | | 0.448 | 0.000 |
| P78324 | Tyrosine-protein phosphatase non-receptor type substrate 1 | | 0.487 | 0.000 |
| Q5W0C9 | Torsin-3A | | 4.092 | 0.000 |
| P35542 | Serum amyloid A-4 protein | | 0.415 | 0.000 |
| Q12841 | Follistatin-related protein 4 | | 6.095 | 0.000 |
| Q08AL8 | Disintegrin and metalloproteinase domain-containing protein 22 | | 0.159 | 0.000 |
| Q96E20 | CD99 antigen-like protein 2 | | 0.074 | 0.000 |
| Q86TY3 | Uncharacterized protein C14orf37 | | 0.164 | 0.000 |
| P13473 | Lysosome-associated membrane glycoprotein 2 | | 4.857 | 0.000 |
| Q92520 | Protein FAM3C | | 2.106 | 0.000 |
| P02763 | Alpha-1-acid glycoprotein 1 | | 3.830 | 0.000 |
| P04075 | Fructose-bisphosphate aldolase | | 0.280 | 0.000 |
| P01303 | Pro-neuropeptide Y | | 0.383 | 0.000 |
| Q9BYH1 | Seizure 6-like protein | | 0.328 | 0.000 |
| Q96FE7 | Phosphoinositide-3-kinase-interacting protein 1 | | 2.771 | 0.000 |
| P22105 | Tenascin-X | | 0.161 | 0.000 |
| Q86UN3 | Reticulon-4 receptor-like 2 | | 0.178 | 0.000 |
| O43493 | Trans-Golgi network integral membrane protein 2 | | 0.166 | 0.000 |
| P09603 | Macrophage colony-stimulating factor 1 | | 0.168 | 0.000 |
| P61278 | Somatostatin | | 0.169 | 0.000 |
| P01344 | Insulin-like growth factor II | | 4.431 | 0.000 |
| Q16620 | BDNF/NT-3 growth factors receptor | | 3.463 | 0.000 |
| P05090 | Apolipoprotein D | | 2.297 | 0.000 |
| P08294 | Extracellular superoxide dismutase [Cu-Zn] | | 2.742 | 0.000 |
| P43251 | Biotinidase | | 3.450 | 0.000 |
| Q580Q6 | EGF-containing fibulin-like extracellular matrix protein 1 | | 3.156 | 0.000 |
| P07602 | Prosaposin | | 7.700 | 0.000 |
| P14735 | Insulin-degrading enzyme | | 8.834 | 0.000 |
